# Emergence and characterization of new OXA-181 variants in France: OXA-1181, OXA-1201, OXA-1205, OXA-1207, and OXA-1226

**DOI:** 10.1128/spectrum.00489-26

**Published:** 2026-04-10

**Authors:** Léa Bientz, Réva Nermont, Cécile Emeraud, Thierry Naas, Rémy A. Bonnin, Laurent Dortet

**Affiliations:** 1 Faculty of Medicine, Team "Resist," UMR1184 "Immunology of Viral, Auto-Immune, Hematological and Bacterial Diseases (IMVA-HB)," INSERM, Paris-Saclay University 89691 , Le Kremlin-Bicêtre, France; 2 Bacteriology Department, Bordeaux University Hospital, Bordeaux, France; 3 Microbiologie Fondamentale et Pathogénicité, UMR 5234, MFP, ARMYNE Team, Université de Bordeaux, CNRS 27051, Bordeaux, France; 4 SEPSIS Comprehensive Center—IHU SEPSIS, Paris, France; 5 French National Reference Center for Antibiotic Resistance: Carbapenemase-Producing Enterobacterales, Hôpital Bicêtre, AP-HP Paris-Saclay 378965 , Le Kremlin-Bicêtre, France; 6 Bacteriology-Hygiene Unit, Assistance Publique-Hôpitaux de Paris, AP-HP Paris Saclay, Bicêtre Hospital 378965 , Le Kremlin-Bicêtre, France; University of Pretoria, Pretoria, Gauteng, South Africa

**Keywords:** enterobacterales, carbapenemases, OXA-48-like variants, *Escherichia coli*, ST410, high-risk clone

## Abstract

Since 2022, the diversity of OXA-48 variants has increased in France. We analyzed the dissemination of five recent variants (OXA-1181, OXA-1201, OXA-1205, OXA-1207, and OXA-1226) mostly identified on plasmids in high-risk *Escherichia coli* clones. Three of them (OXA-1181, OXA-1205, and OXA-1207) displayed the same Ser244Trp substitution with no significant impact on the β-lactams hydrolytic profile.

**IMPORTANCE:**

This study described the recent emergence of five variants (OXA-1181, OXA-1201, OXA-1205, OXA-1207, and OXA-1226) in France. These variants were mostly identified in *Escherichia coli* high-risk clones. In addition, most of these new OXA-48 variants (OXA-1181, OXA-1205, and OXA-1207) were derived, interestingly, from OXA-181, OXA-232, and OXA-484 variants due to the apparition of the same S244W substitution, a residue that delimits the active site cavity. Together, these results threaten the dissemination of these OXA-48 variants in the community, highlighting the need for continuous monitoring to better understand their epidemiology and dissemination.

## OBSERVATION

The spread of carbapenemase-producing Enterobacterales represents a critical public health concern worldwide (1). Since their first identification in 2004, OXA-48–type carbapenemases have become one of the most common carbapenemase families in Western Europe and have disseminated worldwide (2, 3). In France, since 2012, an increasing diversity of OXA-48-like variants with the particular dissemination of OXA-181, OXA-232, OXA-244, and OXA-484 has been reported (4–6). Among them, the most recent OXA-48 variants (OXA-232, OXA-244, and OXA-484) display a critical substitution in the active site of the enzyme (R214S or R214G), resulting in altered enzymatic activity that complicates their detection (7).

This study reports the identification of five new OXA-48-like variants collected from clinical Enterobacterales isolates in France from 2022 to 2025, including three variants sharing the same substitution S244W. We aimed to characterize the molecular epidemiology, antimicrobial resistance profiles, and genetic environments of these new OXA-48-like variants and to investigate the functional impact of this shared S244W substitution.

**Fig 1:**
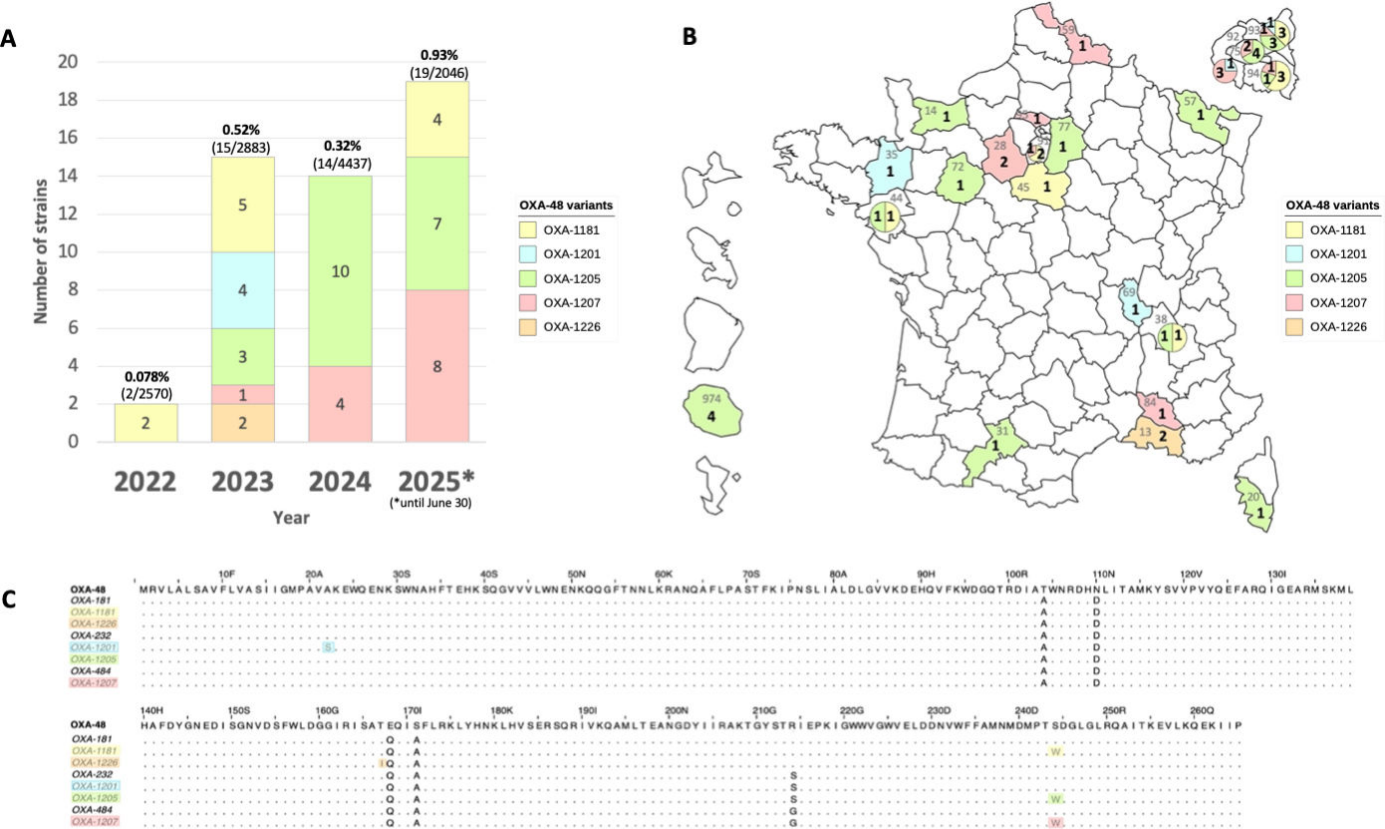
Molecular and epidemiological features of emerging OXA-48-like variants in France, 2022–2025. (**A**) Evolution of five new OXA-48-like–producing Enterobacterales received at the National Reference Center, France, 2022 to 30 June 2025. At the top of the diagram, the first number corresponds to new variants (OXA-1181, OXA-1201, OXA-1205, OXA-1207, and OXA-1226), the second one corresponds to all OXA-48-like variants collected each year. The percentage of new variants among all OXA-48-like producers is indicated in bold. (**B**) Geographical distribution of OXA-48-like variants in France. French department numbers are shown in gray. The number of strains isolated is indicated in bold. Each OXA-48 variant is represented by a distinct color. (**C**) Multiple sequence alignment of OXA-48- and OXA-48-like variants. For each new variant (OXA-1181, OXA-1205, OXA-1207, and OXA-1226), colored boxes indicate amino acid substitutions that are different compared to OXA-181, OXA-232, or OXA-484.

From 1 January 2022 to 30 June 2025, the French National Reference Center for Antimicrobial Resistance (F-NRC) identified five new OXA-48-like carbapenemases named OXA-1181, OXA-1201, OXA-1205, OXA-1207, and OXA-1226. These five variants correspond to point substitution variants of OXA-181, and interestingly, three of them, OXA-1181, OXA-1205, and OXA-1207, displayed a common substitution S244W (Fig. 1C).

During this period, 50 new OXA-48-like variant-producing Enterobacterales have been received by the F-NRC, including 45 *Escherichia coli* (11 OXA-1181, 19 OXA-1205, 13 OXA-1207, and 2 OXA-1226 enzymes) and 5 *Klebsiella pneumoniae* (4 OXA-1201 and 1 OXA-1205; Fig. 1A; Table S1). These five variants represent less than 1% of all OXA-48-like but slowly increased each year (Fig. 1A). By comparison, in 2022, OXA-48-like variants were predominantly OXA-48 (75%), followed by OXA-181 (15%), OXA-244 (10%), OXA-232 (3%), and OXA-484 (2%; data not shown). The isolates were cultured mainly from rectal swabs (*n* = 43) but also from clinical samples, including urine (*n* = 3), blood cultures (*n* = 2), peritoneal fluid (*n* = 1), and vaginal swabs (*n* = 1). Eleven isolates (22%) were recovered from patients who traveled back from Africa (including five from Senegal), eight (16%) from Asia (including seven from India), and one from Turkey (Table S1). All these new variants were accurately detected as OXA-48-like carbapenemases using lateral flow immunochromatographic assays NG-test CARBA-5 (NG Biotech, Guipry, France). We performed short-read whole-genome sequencing on all isolates (Supplemental methods).

**Fig 2:**
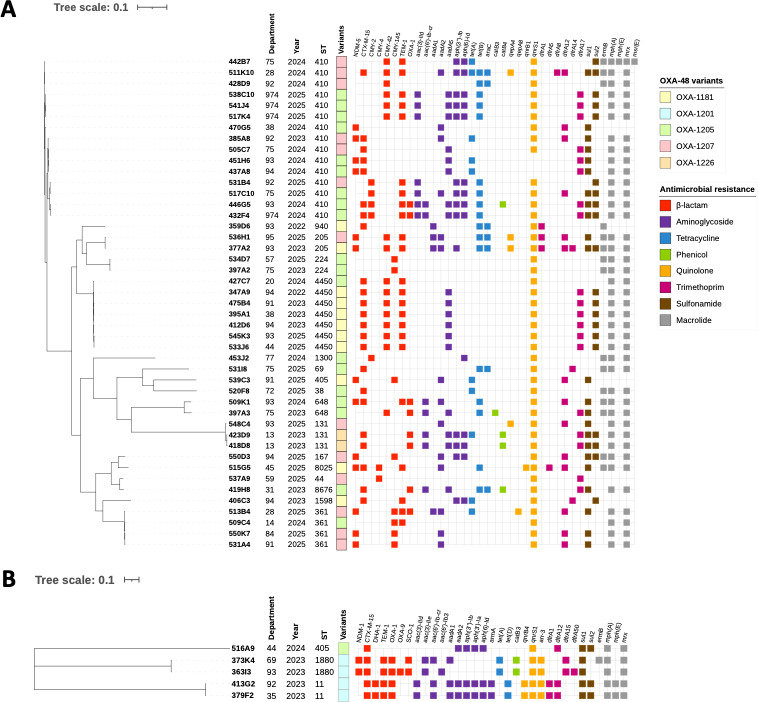
Single-nucleotide polymorphism (SNP)-based phylogenetic tree of the 45 *Escherichia coli* strains (**A**) and 5 *Klebsiella pneumoniae* strains (**B**). For each strain, the department of origin and year of isolation are shown, along with sequence type (ST), OXA-48 variant, and the associated antimicrobial resistance genes (displayed in color according to antibiotic class). The SNP analysis was conducted on a consensus genome covering 67.7%, with strain 347A9 as the reference (**A**) and 82.0%, with strain 363I3 as the reference (**B**). The scale bar indicates the number of substitutions per site. Figures were generated using the Interactive Tree of Life (iTOL) tool.

The OXA-1201 (*n* = 4) and OXA-1226 (*n* = 2) variants remained rare (Fig. 1A), corresponding to small clonal disseminations in 2023 (Fig. 2). OXA-1201 was found exclusively in *K. pneumoniae* isolates, with four strains identified across four different French departments, comprising two sequence types (ST11 and ST1180; Fig. 1B). The two OXA-1201-producing *K. pneumoniae* ST11 were clonally related and co-produced an extended-spectrum β-lactamase (ESBL) CTX-M-15 and an acquired cephalosporinase DHA-1 (Fig. 2B). The two OXA-1201-producing *K. pneumoniae* ST1180 were also clonally related and co-produced an NDM-1 and CTX-M-15 (Fig. 2B). The two OXA-1226-producing *E. coli* were of the ST131 high-risk clones (8) co-producing CTX-M-15 and were clonally related (Fig. 2A).

More consistently over the years, 11 OXA-1181-producing *E. coli* were detected in six different French departments (Fig. 1A and 2A). Six different STs were identified, with ST4450 being the most prevalent, including six closely related (<28 single-nucleotide polymorphisms [SNPs]) isolates co-producing CTX-M-15 and CMY-42 (Fig. S1A).

Interestingly, a polyclonal emergence of OXA-1205 and OXA-1207-producing *E. coli* was observed since 2022 (Fig. 1A). OXA-1205 and OXA-1207 producers were widely distributed across France including metropolitan territory and in French overseas department (i.e., La Réunion island; Fig. 1B). These two variants were mostly (32/33, 97.0%) produced by *E. coli* belonging to 13 different STs including 5 STs considered as high-risk clones: ST410 (*n* = 15), ST361 (*n* = 4), ST648 (*n* = 2), ST38 (*n* = 1), and ST131 (*n* = 1; Fig. 2A) (9). The most prevalent clone of *E. coli*, ST410, remained diverse with the dissemination of at least 10 genetically unrelated isolates (>50 SNPs). Four outbreaks involving nine closely related isolates (≤12 SNPs) have been identified (Fig. S1B). Of note, OXA-1205 was also identified in one ST405 *K. pneumoniae* (Fig. 2B).

These new variants encoding genes were located on ColKP3, ColKP3-IncX3, or ColKP3-IncFII plasmids as described for their respective precursors (2, 3) (Table S1). The genetic environment surrounding these genes was flanked by insertion sequences (IS) with ΔIS*Ecp1* element upstream, providing the promoter region necessary for gene expression. Three genetic backgrounds were identified: (i) in most of the cases (43/50), as described previously for *bla*_OXA-181_ and *bla*_OXA-484_ (4), *bla*_OXA-181-like_ gene was flanked by IS*3000* upstream the ΔIS*Ecp1* and Δ*lysR*, Δ*ere*, a truncated replicase encoding gene and IS*Kpn19*, downstream; (ii) *bla*_OXA-1207_ (strain 548C4) in the same genetic organization but lacking the truncated replicase encoding gene; and (iii) *bla*_OXA-1205_ (strains 437A8 and 451H6) associated with a different IS*6* family element downstream (Fig. S2). Genetic contexts (ii) and (iii) were not found in the NCBI database. The four *bla*_OXA-1201_ genes were located on a 6,141 pb ColKP3-type plasmid, as previously described for *bla*_OXA-232_ (5).

For all isolates, minimum inhibitory concentrations (MICs) were determined by broth microdilution using the Sensititre customized plates (Thermo Fisher Scientific). As mentioned previously, all 50 isolates co-produced other β-lactamases, except for one strain that expressed only OXA-1207 (strain 548C4). The most frequent β-lactamases were NDM-5 (*n* = 13), CTX-M-15 (*n* = 28), and CMY-42 (*n* = 16; Fig. 2). Due to the presence of ESBL or acquired cephalosporinases, these clinical isolates were resistant to third and fourth generation cephalosporins and to aztreonam. As expected, ceftazidime-avibactam and cefepime-enmetazobactam remained effective against isolates that did not co-produce NDM, whereas aztreonam–avibactam was the most effective treatment option for NDM co-producers. However, decreased susceptibility (MIC = 4 mg/L) or resistance to aztreonam-avibactam was observed in 24 *E. coli* clinical isolates belonging to eight different STs, including seven ST410, seven ST4450, and four ST361, all harboring either a YRIK, YRIN, or YRIP insertion in their penicillin-binding protein 3 (PBP3), the main target of aztreonam. All strains remained susceptible to colistin and eravacycline (Table S1).

To determine the impact of S244W amino-acid substitutions on β-lactams MICs, the *bla*_OXA-1181_, *bla*_OXA-1205_, and *bla*_OXA-1207_ genes as well as their respective precursors (*bla*_OXA-181_, *bla*_OXA-232_, and *bla*_OXA-484_ genes) were cloned into pCR-Blunt II-TOPO (Invitrogen) using the primers preOXA-48A (5′-TATATTGCATTAAGCAAGGG-3′) and preOXA-48B (5′-CACACAAATACGCGCTAACC-3′) and electroporated into *E. coli* TOP10 as described previously (7, 10, 11). Overall, the S244W substitution had no significant impact on the MICs of the tested β-lactams (Table 1).

**TABLE 1:** Effect of Ser244Trp substitution on β-lactam antibiotics susceptibility^*a*^

		MIC (mg/L)					
		*E. coli* TOP10 pTOPO-					
β-lactam	*E. coli* TOP10	OXA-181	OXA-1181 (OXA-181 S244W)	OXA-232	OXA-1205 (OXA-232 S244W)	OXA-484	OXA-1207 (OXA-484 S244W)
Ceftazidime	0.5	0.5	0.5	0.25	0.5	0.5	1
Ceftazidime-avibactam	0.25	0.25	0.25	0.25	0.25	0.25	0.5
Aztreonam	0.12	≤0.06	0.12	≤0.06	0.25	0.12	0.12
Aztreonam-avibactam	≤0.06	0.12	≤0.06	≤0.06	≤0.06	≤0.06	0.12
Cefepime	≤0.06	0.12	≤0.06	≤0.06	≤0.06	≤0.06	0.12
Cefepime-enmetazobactam	≤0.06	≤0.06	≤0.06	≤0.06	≤0.06	≤0.06	≤0.06
Cefepime-taniborbactam	≤0.06	≤0.06	≤0.06	≤0.06	≤0.06	≤0.06	≤0.06
Cefepime-zidebactam	≤0.06	≤0.06	≤0.06	≤0.06	≤0.06	≤0.06	≤0.06
Ceftolozane-tazobactam	≤0.25	0.5	0.5	0.5	0.5	2	4
Ertapenem	≤0.06	0.5	0.5	0.5	0.5	0.5	0.25
Imipenem	0.25	0.5	0.5	0.25	0.25	0.25	0.25
Imipenem-relebactam	0.25	0.25	0.25	0.25	0.25	0.25	0.25
Meropenem	≤0.06	0.12	0.25	≤0.06	0.12	0.12	≤0.06
Meropenem-vaborbactam	≤0.06	0.12	0.12	≤0.06	≤0.06	0.12	≤0.06
Temocillin	16	>128	128	32	64	64	128

^*a*^ MIC, minimum inhibitory concentration.

The diversification of OXA-48-like carbapenemases is progressively increasing in France, illustrating a dynamic evolutionary process. Since 2022, five new variants were identified at the F-NRC. While OXA-1201 and OXA-1226 variants remained sporadic, possibly resulting from random point mutations, the OXA-1181, OXA-1205, and OXA-1207 variants were characterized by the recurrent S244W substitution that occurred independently in three OXA-181-like variants: OXA-181, OXA-232, and OXA-484. These new variants have been identified predominantly in *E. coli* of various STs, particularly enriched in the high-risk STs such as ST410 (4, 6, 8), ST361 (12), and ST167 (9) as observed with other carbapenemases (OXA-244, OXA-484, and NDM-5). These new OXA-181-like variants were mostly co-expressed with NDM, CTX-M, or CMY enzymes, which facilitated their detection through their high resistance profiles. Of note, all these new variants were accurately detected as OXA-48-like carbapenemases using lateral flow immunochromatographic assays performed on bacterial colonies. The encoding genes were located on plasmids showing high coverage and close identity to previously described ColKP3, ColKP3-IncX3, or ColKP3-IncFII-type, even though complete plasmid sequencing was not performed using long-read technology. These plasmids enable their transmission by conjugation and facilitate their dissemination. To the best of our knowledge, this represents the first report of the emergence of five novel variants from 2022 in a single country. Until now, only one study from Qatar has reported an OXA-1207-producing *E. coli* ST617 in 2025 (13). Nevertheless, analysis of public genomes in the NCBI Pathogen database (Table S2) showed that the *bla*_OXA-1201_ and *bla*_OXA-1226_ genes were only found in our study, but the three other variants have been detected in 209 *E. coli*, 9 *K. pneumoniae*, 2 *Citrobacter freundii*, and 1 *Enterobacter hormaechei* genomes worldwide. More specifically, 109 isolates producing OXA-1207 were identified across 11 countries, mainly in the USA (*n* = 50) and India (*n* = 37), but also in Canada, Singapore, Germany, the Netherlands, Senegal, Vietnam, the UK, Qatar, and Ireland. In addition, 98 isolates producing OXA-1205 were isolated in seven countries (USA [*n* = 49], India [*n* = 37], the UK, Canada, Ireland, the Netherlands, and Australia). Finally, 14 isolates producing OXA-1181 were found across seven countries (Canada, India, USA, Australia, Bangladesh, New Zealand, and Tanzania). This finding highlights the global distribution of the OXA-1205 and OXA-1207 variants and suggests that epidemiological investigations in other countries and further studies on the hydrolytic activity of these enzymes should be performed. Although cloning experiments did not highlight any significant effect of the S244W substitution on tested β-lactams MICs, the independent occurrence of this substitution in OXA-181, OXA-232, and OXA-484 is particularly intriguing. Indeed, the Ser-244 delimits the active site cavity with Ile-102, Gln-124, and Arg-214 (14, 15), but the predicted structural arrangement of Trp-244 remains hypothetical (Fig. S3). Determining how the tryptophan residue could alter the active site configuration is crucial and requires further investigations through biochemical and structural characterization by kinetic and crystallography analysis.

In conclusion, our study described the emergence of OXA-1181, OXA-1205, and OXA-1207 in France and worldwide, highlighting the need for continuous monitoring to better understand their epidemiology and dissemination.

## Data Availability

The sequencing data of isolates have been deposited in the NCBI under BioProject accession number PRJNA1356282.

## SUPPLEMENTAL MATERIAL

Supplemental material spectrum.00489-26-s0001.pdf Fig. S1; Supplemental methods.

Table S1 spectrum.00489-26-s0002.xlsx Epidemiological and genetic characteristics of OXA-1181, OXA-1201, OXA-1205, OXA-1207, and OXA-1226-producing Enterobacterales in France, French National Reference Center, 2022–2025.

Table S2 spectrum.00489-26-s0003.xlsx Isolates producing OXA-1181, OXA-1201, OXA-1205, OXA-1207, and OXA-1226 from the public genome of the NCBI Pathogen Detection Database.

ASM does not own the copyrights to Supplemental Material that may be linked to, or accessed through, an article. The authors have granted ASM a non-exclusive, world-wide license to publish the Supplemental Material files. Please contact the corresponding author directly for reuse.
