# Supplementary material for: Emergence and characterization of new OXA-181 variants in France: OXA-1181, OXA-1201, OXA-1205, OXA-1207, and OXA-1226
Source: Microbiol Spectr. 2026 Apr 10;14(5):e00489-26. doi: 10.1128/spectrum.00489-26 (PMC13141865; doi:10.1128/spectrum.00489-26)
Supplement: Supplemental material — Fig. S1; Supplemental methods. [file spectrum.00489-26-s0001.pdf]

SUPPLEMENTAL MATERIAL

Fig. S1. Phylogenetic tree with SNPs matrix (single-nucleotide polymorphism) for OXA-48-like–producing *Escherichia coli* belonging to ST410 (A) and ST4450 (B).

For each strain, the department of origin and year of isolation are shown, along with sequence type (ST), OXA-48 variant, and the associated antimicrobial resistance genes (displayed in color according to antibiotic class). The SNP analysis was conducted on a consensus genome covering 88.6%, with strain 385A8 as the reference (A) and 94.0%, with strain 347A9 as the reference (B). In the SNP matrix, a threshold of 20 SNPs was chosen to define isolates as belonging to the same cluster. The scale bar indicates the number of substitutions per site. Figures were generated using the Interactive Tree of Life (iTOL) tool.

Fig. S2. Genetic background of *bla*$_{\text{OXA-1181, -1205, -1207, -1226}}$ genes

The *bla*$_{\text{OXA-48-like}}$ genes are highlighted in red, mobile genetic elements in blue, and other genes in yellow. Regions sharing >99% sequence identity are indicated in light grey.

Fig S3. Structural model of OXA-181 variants.

The three Class D β-lactamase motifs are indicated as sticks: motif I (Ser70, Thr71, Phe72, Lys73; blue), motif II (Ser118, Val119, Val120; magenta), motif III (Lys208, Thr209, Gly210; green). The Ω-loop is shown in orange. Residues Gln124 (yellow), Ile102 (yellow), Arg214 and Ser244 delimit the active site cavity. Variant-specific residues are marked at position 214 (Arg/Ser/Gly; black) and 244 (Ser/Trp; red). AlphaFold3 modeling visualized in PyMOL.

Supplementary Method

Fig S1. Phylogenetic tree with SNPs matrix (single-nucleotide polymorphism) for OXA-48-like–producing *Escherichia coli* belonging to ST410 (A) and ST4450 (B).

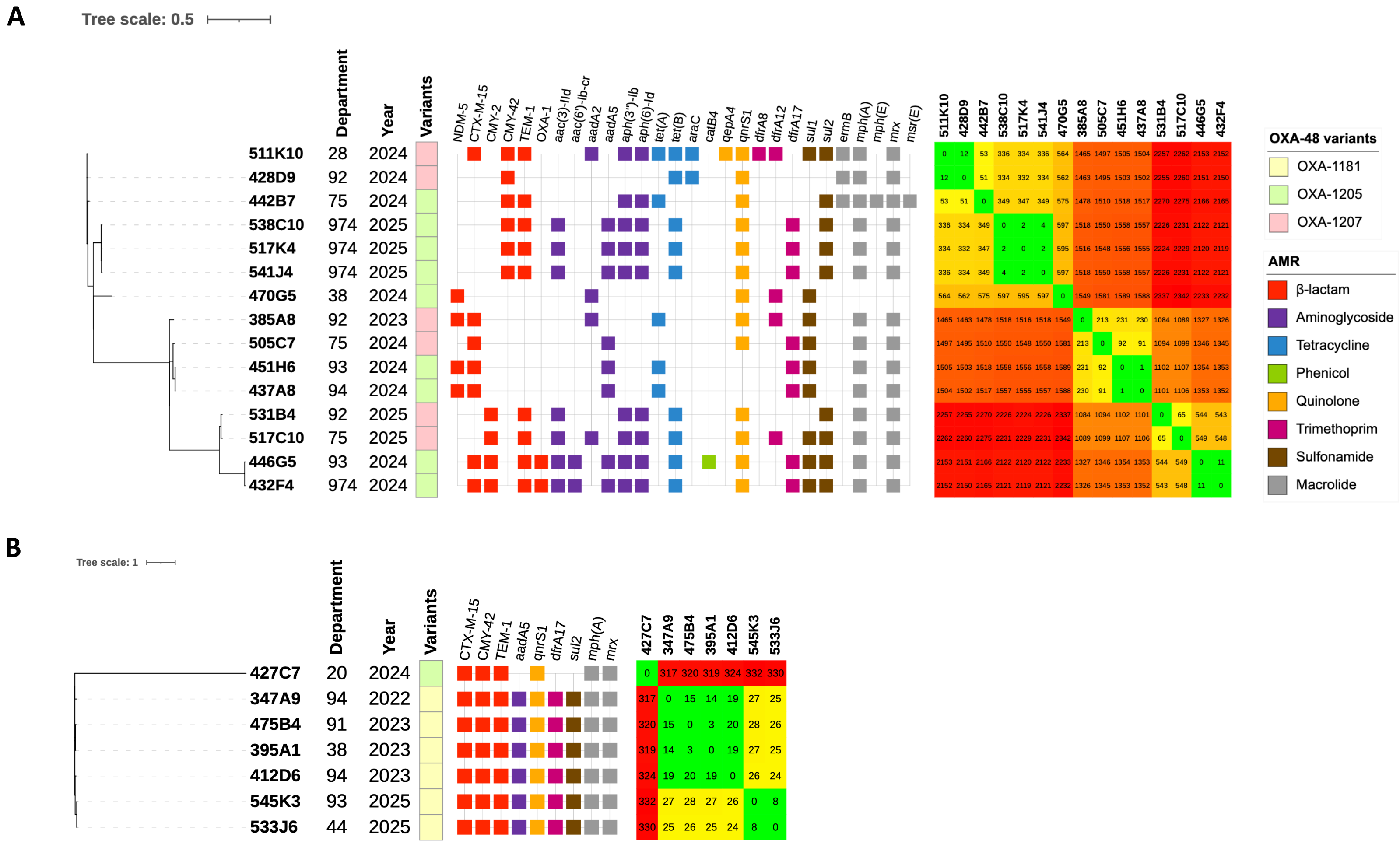

For each strain, the department of origin and year of isolation are shown, along with sequence type (ST), OXA-48 variant, and the associated antimicrobial resistance genes (displayed in color according to antibiotic class). The SNP analysis was conducted on a consensus genome covering 88.6%, with strain 385A8 as the reference (A) and 94.0%, with strain 347A9 as the reference (B). In the SNP matrix, a threshold of 20 SNPs was chosen to define isolates as belonging to the same cluster. The scale bar indicates the number of substitutions per site. Figures were generated using the Interactive Tree of Life (iTOL) tool.

Fig. S2. Genetic background of *bla*$_{OXA-1181, -1205, -1207, -1226}$ genes

The *bla*$_{OXA-48-like}$ genes are highlighted in red, mobile genetic elements in blue, and other genes in yellow. Regions sharing >99% sequence identity are indicated in light grey.

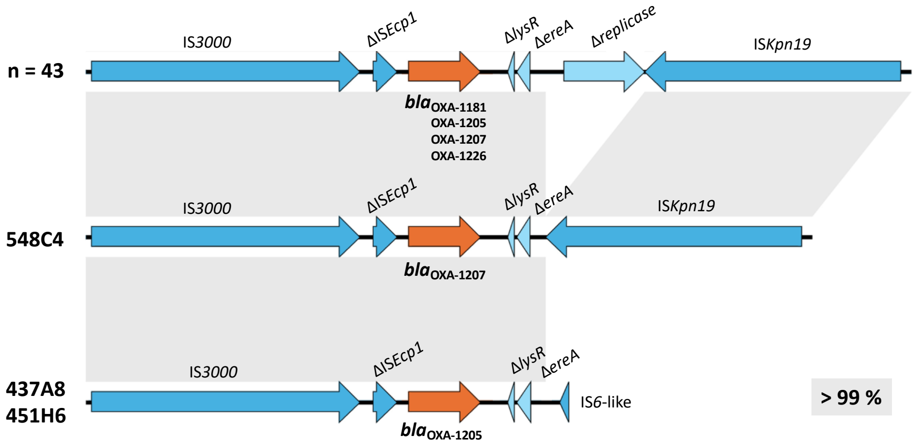

Fig S3. Structural model of OXA-181 variants.

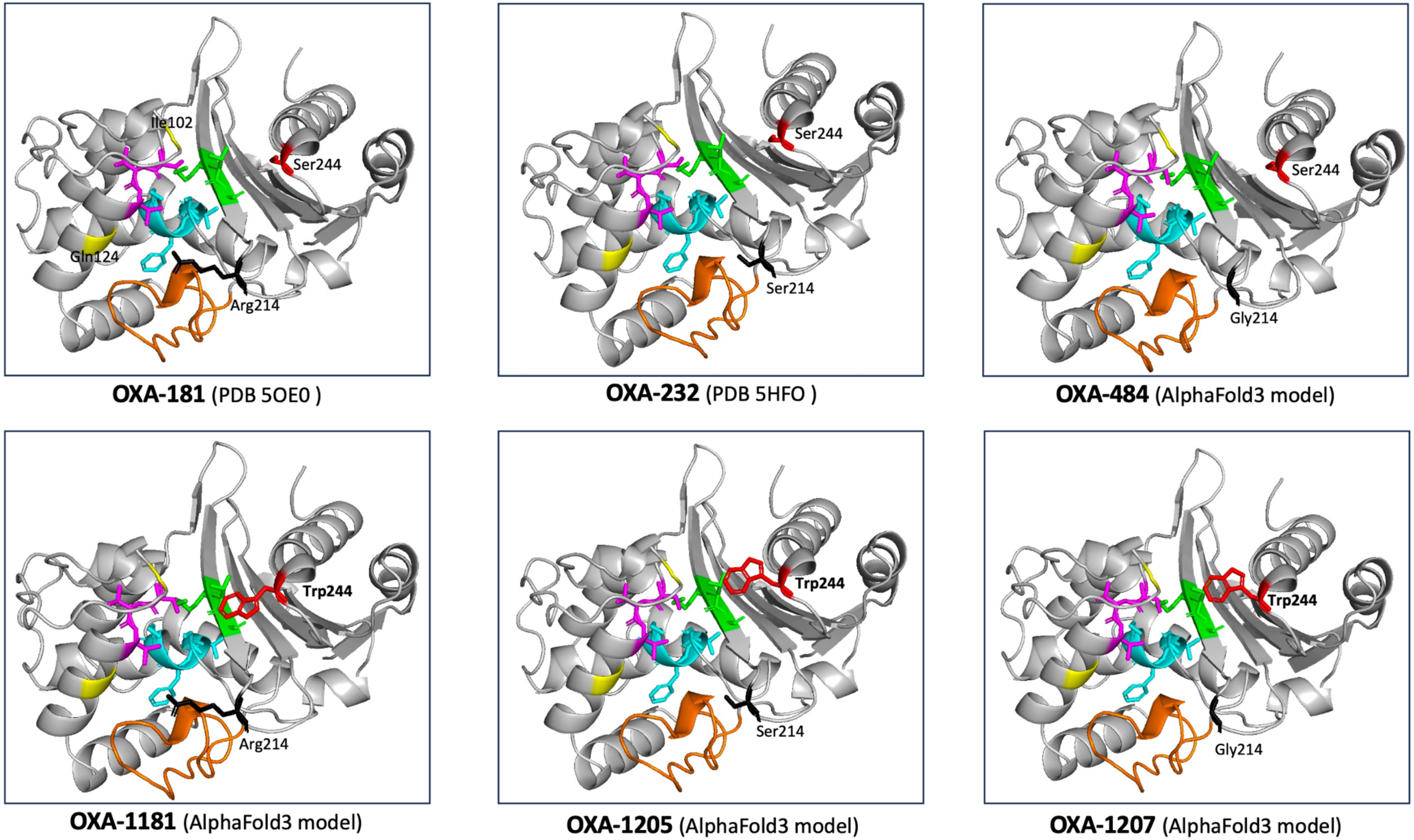

The three Class D β-lactamase motifs are indicated as sticks: motif I (Ser70, Thr71, Phe72, Lys73; blue), motif II (Ser118, Val119, Val120; magenta), motif III (Lys208, Thr209, Gly210; green). The Ω-loop is shown in orange. Residues Gln124 (yellow), Ile102 (yellow), Arg214 and Ser244 delimit the active site cavity. Variant-specific residues are marked at position 214 (Arg/Ser/Gly; black) and 244 (Ser/Trp; red). AlphaFold3 modeling visualized in PyMOL

Supplementary Methods.

Short-read whole-genome sequencing was performed on all isolates using Illumina HiSeq technology (https://www.illumina.com; BioProject accession no. PRJNA1356282). Reads were assembled with Shovill version 4.0.0 (https://github.com/tseemann/shovill) and SPAdes version 4.0.0 (https://github.com/ablab/spades). Multilocus sequence typing and resistome characterization were conducted using the pubMLST (https://pubmlst.org) and ResFinder 4.7.2 (http://genepi.food.dtu.dk/resfinder) databases. Plasmid replicon types were identified using the PlasmidFinder (https://cge.food.dtu.dk/services/PlasmidFinder). Short-read data were mapped to reference plasmids carrying *bla*$_{\text{OXA-181-like}}$ gene using CLC Genomics Workbench (Qiagen) allowing determination of read coverage (%) and identity (%). Single nucleotide polymorphisms (SNPs) were detected using the CSI Phylogeny pipeline (https://www.genomicepidemiology.org) with default parameters using the oldest strain as the reference. In the SNP matrix, a threshold of 20 SNPs was chosen to define isolates as belonging to the same cluster, based on what is described in the literature for outbreak-oriented relatedness thresholds. The resulting phylogenetic trees were visualized and annotated with Interactive Tree of Life (https://itol.embl.de). Structural models of OXA-181 variants were predicted using AlphaFold3 (1) and visualized with PyMOL software with Class D β-lactamase motifs highlights (2).

1. Abramson J, Adler J, Dunger J, Evans R, Green T, Pritzel A, Ronneberger O, Willmore L, Ballard AJ, Bambrick J, Bodenstein SW, Evans DA, Hung C-C, O'Neill M, Reiman D, Tunyasuvunakool K, Wu Z, Žemgulytė A, Arvaniti E, Beattie C, Bertolli O, Bridgland A, Cherepanov A, Congreve M, Cowen-Rivers AI, Cowie A, Figurnov M, Fuchs FB, Gladman H, Jain R, Khan YA, Low CMR, Perlin K, Potapenko A, Savy P, Singh S, Stecula A, Thillaisundaram A, Tong C, Yakneen S, Zhong ED, Zielinski M, Žídek A, Bapst V, Kohli P, Jaderberg M, Hassabis D, Jumper JM. 2024. Accurate structure prediction of biomolecular interactions with AlphaFold 3. Nature 630:493–500.

2. Docquier J-D, Calderone V, De Luca F, Benvenuti M, Giuliani F, Bellucci L, Tafi A, Nordmann P, Botta M, Rossolini GM, Mangani S. 2009. Crystal Structure of the OXA-48 β-Lactamase Reveals Mechanistic Diversity among Class D Carbapenemases. Chemistry & Biology 16:540–547.
